# Dental-Dam for Infection Control and Patient Safety during Clinical Endodontic Treatment: Preferences of Dental Patients

**DOI:** 10.3390/ijerph15092012

**Published:** 2018-09-14

**Authors:** Ahmad Madarati, Seema Abid, Faisal Tamimi, Ali Ezzi, Aya Sammani, Mohamad Bachar Abou Al Shaar, Muhammad Zafar

**Affiliations:** 1 Department of Restorative Dentistry, College of Dentistry, Taibah University, Al Madinah, Al Munawwarah 41311, Saudi Arabia; amadarati@taibahu.edu.sa (A.M.); drseemabid@yahoo.com (S.A.); ftamimi@taibahu.edu.sa (F.T.); ezzi202@gmail.com (A.E.); ayatgod@gmail.com (A.S.); 2 Department of Substitutive Restorative Dental Sciences, Faculty of Dentistry, Aleppo University, Aleppo, Syria; 3 Department of Preventive Dental Sciences, College of Dentistry, Taibah University, Al Madinah, Al Munawwarah 41311, Saudi Arabia; doctorimplant33@gmail.com; 4 Department of Dental Materials, Islamic International Dental College, Riphah International University, Islamabad 44000, Pakistan

**Keywords:** root canal, clinical endodontics, dental pulp, general practice, restorative dentistry

## Abstract

Background: A number of factors (first experience, treating clinician and time to place dental-dam) may influence patients’ preferences regarding dental-dams. In general, patients accept placing it and that it must be used for teeth isolation during endodontic procures for the sake of patient safety and infection control. Objectives: The aim of this study is to investigate preferences and experiences of patients using dental-dam (DD) isolation during root canal treatment (RCT) and to explore influencing factors among the residents of Madinah Munnawara, Saudi Arabia. Methods: Following an ethical approval and a pilot study, a self-administrated questionnaire was distributed to 305 patients attending endodontic clinics at the Taibah University College of Dentistry (TUCOD) over six months. Patients voluntarily participated in the study after understanding the methodologies and signing a consent form. They were asked to fill out a questionnaire on their experiences and preferences in placing the DD during RCT. Data were analyzed using the Chi-square test at *p* = 0.05. Results: The response rate was 91%. There was no significant correlation between patients’ preferences and their race, age and gender (*p* > 0.05). The majority of participants (74.3%) would prefer to use a DD in their next session (*p* < 0.001). This preference negatively correlated with the time required to place a DD and the duration of the current visit (*p* < 0.001). While most of those who would prefer to use a DD in their next visit were pleased with how it was placed in the current session (76.6%), most of those who would not do so (66.7%) were uncomfortable. Overall, the highest proportion of participants (40.2%) reported that prevention of instrument swallowing was the most important advantage of DD isolation (*p* < 0.001). Conclusions: Overall, DD isolation for RCT is generally well accepted by patients regardless of their country of origin, gender, education and awareness of its advantages. Patients’ safety was the most attractive advantage for patients to the application of the DD. The time required to place the dental dam and first visit experience in placing the DD affect patients’ future preference.

## 1. Introduction

The dental-dam (DD) has been an ideal tool for tooth isolation and a standard of care in dentistry, especially during root canal treatments (RCT) [1]. Historically, Barnum introduced the concept of DD isolation to dental professionals in 1864 [2]. An aseptic operating field by isolation from oral and salivary contamination is a great and significant advantage of the DD for both the operator and the patient during endodontic treatment [3]. It also allows for safe insertion of inter-appointment medicaments necessary to disinfect the root canal system [4,5]. From patient as well as clinician perspectives, two major aspects are essential; patients’ safety and cross-infection control. The dental dam is a fundamental tool that protects patients by preventing inhalation or ingestion of endodontic instruments [6] and retracting soft tissues [7]. Unfortunately, there are many reports of inhalation or ingestion of endodontic instruments during root canal treatment as a result of not considering DD isolation [6,7,8,9,10,11,12,13].

In addition, the DD promotes better cross-infection control for both patients and the dental team [3], especially in cases of communicable diseases such as acquired immune deficiency syndrome (AIDS) and viral hepatitis. The dentists and dental assistants are protected against infections which can be transmitted by the patient’s saliva. A previous study reported that using a DD is an excellent barrier to the potential spread of infectious disease in the dental office [3].

Despite these advantages, DD isolation during root canal treatment is still not adopted in dental practice in many countries [14,15,16,17]. The major barriers of using DDs include: challenging placement techniques, time consuming (from a dentist’s point of view), lack of training during undergraduate training, and cost of equipment and materials [16,18,19,20,21]. In addition, patient discomfort and rejection have been proposed as barriers for using DDs [16,17,19,22,23]. However, a few reports showed that patients have no objection to DD application and they prefer having it placed in future visits [24,25]. 

A recent study has shown slight improvement in using DDs in Saudi dental practices [17] compared to a previous report [26]. However, there is no study on patient perception regarding the importance of using DD isolation as a tool for better cross-infection control and safe endodontic procedures. In addition, there has been no reports on patient preference on placing DDs during RCTs. Hence, there is a need to investigate these important aspects within the Saudi community. This is especially true as the professional environment is not the same in different countries with different cultures. In addition, unlike other countries, Saudi Arabia has a large diversity of patients from different countries, which may have great impact. To our knowledge, the current study was the first to systematically explore, in depth, these factors on patient preference. Therefore, the aim of this study was to investigate opinions, experiences and preferences of patients on placing DDs during RCTs. Specifically, it aimed to estimate the frequency of patient acceptance of placing DDs and to explore affecting factors. The survey mainly aimed to answer the following questions:

□ Would there be any significant difference between patients who would prefer placing a DD in a future visit and those who would not?
□ Could patient acceptance be influenced by factors such as DD application time and length of the treatment session with the DD in place?
□ Which policy do patients prefer (mandatory, optional) to increase the use of DDs in dental practice?

Consequently, the study aimed to test the following null hypothesis: 

There would be no significant differences between the proportion of patients who would prefer using DD in the future and that of patients who would not.

## 2. Materials and Methods 

This observational descriptive study was ethically approved by the Taibah University College of Dentistry Research and Ethics Committee (TUCDREC) and conducted between January and June 2017. A pilot self-administrated questionnaire (Appendix A) was distributed to a group of patients that underwent RCTs in the clinics at the TUCOD to ensure that the questions were easily understood and that all relevant aspects were included. Convenient sample size over a period of six months was determined, taking into consideration the sample sizes of three previous studies (110, 179 and 220 patients) [24,27,28]. This study included the following governmental centers in Madinah Munawara: the TUCOD and King Fahad General Hospital (KFGH). A total of 305 patients were approached for voluntary participation after they underwent a session of a root canal treatment procedure. The investigator explained the aims and methodologies of the study to the patients and confirmed that all information would remain confidential and anonymous. In addition, patients were assured that their decision to complete the survey would not affect the dental service they would receive in the future. Upon acceptance to participate in the study, patients were asked to sign a consent form. They were requested to hand the questionnaire form, upon completion, to a third person, who was not related to the study. Responses were collected, and data were entered into SPSS 20 for Windows software (SPSS Inc., Chicago, IL, USA) and analyzed using the Chi-square test at the 0.05 level of significance. 

## 3. Results

### 3.1. Response Rate and Preference of Using a DD in the Next Session

Out of 305 patients who were invited to the study, 278 patients accepted to complete the survey; resulting in a 91.1% overall response rate. The proportion of participants who would prefer using a DD in the next session (81.6%) was significantly greater than those who would not (18.4%) (*p <* 0.001) (Table 1). There was no significant difference between Saudi and Non-Saudi patients. 

### 3.2. Age and Gender of Participants

The proportion of male participants (45.2%) was not statistically significant from that of females (54.8%) (*p* = 0.115) (Table 2). In addition, the proportion of male participants who would prefer to use a DD in the next visit (79.7%) was not statistically significant from that of females who would do so (83.2%) (*p* = 0.530). Significantly, more patients (31.1%) were 21–30 years old (*p <* 0.001); with no significant difference between males and females (*p* = 0.057). Overall there was no correlation between participant age and their preference in using a DD in the next visit (*p* = 0.07). 

### 3.3. Participant Education

Whilst the highest proportion had a Secondary School degree (48.6%), the lowest had completed University Postgraduate Study (4.4%), followed by University and Primary School degrees (26.9% and 21.3%, respectively). These differences were statistically significant (*p <* 0.001) (Table 2). There was no correlation between participant education level and their preferences in using a DD in the next visit (*p* = 0.090). 

### 3.4. Centre and Clinicians Providing Treatments

The proportion of participants who would prefer using a DD in the next session and were treated at the KFGH (95.7%) was significantly greater than those who would do so and were treated at TUCOD clinics (74.4%) (*p <* 0.001) (Table 3). While the highest proportion of patients at TUCOD were treated by undergraduate students, the highest proportion of patients at KFGH were treated by an endodontic consultant (*p <* 0.001). Patient preference on using a DD in the next visit significantly correlated with classification of the treating clinicians (*p <* 0.001). The vast majority of patients treated by consultants (93.2%) would prefer to use a DD in the next visit, which was significantly greater than those treated by undergraduate or postgraduate students (61.2% and 66.7% respectively).

### 3.5. Advantage of Using a DD

Overall, the highest proportion (44.8%) of patients reported that prevention of instrument inhalation or ingestion is the main advantage of DD use (*p <* 0.001), with no significant differences between the proportion of those who would prefer to use a DD in the next visit (46.8%) and those who would not (39.1%) (*p* = 0.416) (Table 4). The lowest proportion (8.1%, all of them would prefer to use a DD in the next visit) reported that cross-infection control is the main advantage. 

### 3.6. Time Required for Placing the DD

Overall, the highest proportion of patients (72.3%) needed up to one minute to place the DD by the treating clinician (*p <* 0.001), with a significantly greater proportion of those who would prefer to use a DD in the next visit (77.9%) compared to those who would not (52.2%) (*p <* 0.001) (Table 5). Overall, there was a significant correlation between participant preference in using a DD in the next visit and the time taken for placing the DD in the current visit (*p* = 0.001). 

### 3.7. First Visit Experience

Significantly, most participants (65.7%) were pleased with the placing of the DD in the first (current) visit (Table 5). Overall, there were significant differences between those who would prefer to use a DD in the next visit and those who would not (*p <* 0.001). The highest proportion of those who would prefer to use a DD in the next visit were pleased with how it was placed in the current session (76.8%). By contrast, the highest proportion of those who would prefer not to use a DD in the next visit were uncomfortable with the placing of the DD in the current session (70%).

### 3.8. Duration of Current Session with DD in Place

Overall, the highest proportion of patients (67.4%) reported that the duration of the current session with a DD in place was reasonable (*p <* 0.001), with a significantly greater proportion of those who would prefer to use a DD in the next visit (71.2%) compared to those who would not (48%) (*p <* 0.001) (Table 5).

### 3.9. Explanation of Reasons for DD Use

Significantly, the majority of patients (73.1%) reported that the dentist explained the reasons for using a DD (*p <* 0.001) (either in detail (39.9%) or briefly (33.2%) (*p* = 0.189)). However, this did not affect participant preferences on placing a DD in the next visit (*p* = 0.568) (Table 5).

### 3.10. Policy of Using DDs

The highest proportion of patients reported that placing the DD should be optional (up to dentists) (41.2%), followed by those who believe it should be mandatory (33.1%) or optional (up to patients) (23.9%) (Table 6). These differences were statistically significant (*p* = 0.002). Whilst the highest proportion of those who would not prefer placing a DD in the next visit and believed that the DD should be optional (up to patients) (52%), the lowest of those who would prefer placing a DD reported the same policy for DD placement (17.6%) (*p <* 0.001).

## 4. Discussion

Survey studies can provide valuable information about preferences, opinions, attitudes, experiences, and demographics of participants. However, such studies should be carefully conducted, so that results are representative and can be generalized [29,30]. Survey studies have shown diversity of DD usage during RCTs worldwide [14,15,16,17,19,20,21]. DD isolation during RCTs has been considered a standard of care due to its many advantages [1]. However, it is still not being commonly used by dental clinicians because of different claims such as inconvenience to the clinician, increased treatment cost and prolonged treatment time [21]. According to some recent studies, patient discomfort and rejection have been reported as possible restraints [24,25,28]. This is especially true as some aspects, such as community culture and the dental profession environment, vary among different countries and hence may affect patient preferences. In addition, gender, age, race and the education level of patients, as well as the duration of DD placement, may have an impact. Therefore, this study aimed to investigate in depth attitudes, experiences and preferences of patients receiving RCTs in governmental Saudi dental practices. 

The proportion of male participants (45.2%) was not statistically significant from that of females (54.8%), which reflects good study sampling. In addition, there was no significant difference between males and females regarding age groups, although the highest proportion of them (31.1%) were 21–30 years old. There were significant differences among participant level of education. While the highest proportion had a Secondary School education (47%), the lowest proportion of them had a University Postgraduate Studies degree (4.4%). Nevertheless, results showed that patient gender, age, country of origin and education level did not have any significant impact on their preferences on placing DDs. In contrast, Stewardson and McHugh’s [28] results showed that women in one subgroup preferred DD use. Also, Vedavathi et al. [31] showed that female patients had a positive attitude and preferred the use of DDs in the future.

The highest proportion of participants in our study (65%) were pleased with the placing of a DD during the session, after which they completed the questionnaire. These findings are consistent with those obtained in previous studies [25,27,28]. One possible reason is that patients compared the current treatment experience with that of previous RCTs without a DD. Another reason is that patient attitude is dependent to some extent on the experience and skill of the dentist providing the treatment, which in turn leads to faster and more comfortable application of DDs. Previous studies showed that good experience and positive attitudes of the dentists placing the DD and performing the treatment reflects on positive attitudes of patients [24,28]. Our results showed that a relatively short time was required to place a DD (about 1 minute) in 75% of cases. The results also revealed a significant positive correlation between participant preferences in using a DD in the next visit and the time required for placing a DD. This is, however, in contrast to results of previous studies in which the time required to place a DD had no influence on patient preference on future DD use [24,28]. Apart from this discrepancy, it is accepted that a short time required for DD placement usually reflects good dental practice and sufficient experience. It was reported previously that placing a DD, even in the hand of inexperienced dentists, does not take more than few minutes [24,25,32,33]. It could be argued that the good patient acceptance in our study was because patients might have thought that their negative response would have likely affected future treatment eligibility. However, such an assumption can be excluded because patients were assured that their response would not affect their right to receive dental treatment. Furthermore, the majority of patients (82%) preferred placing a DD in the next visit. Moreover, the highest proportion of those who would prefer a DD in the next visit were pleased with how it was placed in the current session (77.7%).

Our results showed that the proportion of participants who would prefer using a DD in the next session and were treated at KFGH (95.7%) was significantly greater than those who would do so and were treated at TUCOD clinics (74.4%). This may confirm our assumption, as indicated earlier, that different treatment environments can affect patient attitudes and preferences. Those treated by undergraduate students were the least likely to prefer using a DD in the next visit. On the other hand, the vast majority of those who were treated by endodontic consultants (93.2%) would prefer using a DD in the next visit. While the highest proportion of patients at TUCOD were treated by undergraduate students, the highest proportion of patients at KFGH were treated by endodontic consultants. However, the clinician providing RCTs may be another environment factor that may affect patient preferences. Therefore, these results should be carefully interpreted, and such a conclusion should be drawn with caution.

It was obvious that the experience of placing a DD in the first session did have an impact on patient overall attitudes. The highest proportion of those who would not prefer to use a DD in the next visit were uncomfortable with the current experience of DD placement. We assumed that a thorough explanation of the advantages of using a DD may positively affect patient preferences. However, the results did not show such an impact whatsoever, with only 27.4% of patients confirming that the dentist did not explain why a DD would be used. One possible reason for such a result is that the majority of patients (81.9%) would prefer to use a DD in the next visit in the first instance. These findings, however, contradict with those of Kapitan et al. [27], where explanation of DD use in detail prior to the procedure had a significant impact on patient preferences. Nevertheless, these results again support the reality that whether a patient had a good experience in the first session most likely determines their preference in using a DD in the next visit.

This study revealed how patient experiences and attitudes towards placing a DD, whether acceptance or rejection, reflected on their opinion regarding the length of the current session time. The majority of those who would prefer to use a DD in the next session (72%) thought that the current session duration was reasonable. By contrast, most of those who would not prefer to use a DD in the next session (52%) thought that the current session was long. These results were inconsistent with those of previous studies where the duration of the treatment while the DD was in place had no influence on patient preferences on DD placement [24,27,28].

Patient safety is usually the first priority in medical and dental fields. The current study reflected this fundamental concept as the highest proportion of participants (46.8%) reported that the most important advantage of using a DD is the prevention of accidental swallowing of files, although this had no significant influence on the patients preferring to use a DD in the next visit. It is likely that patients who were better informed tend to cooperate more, even if they were not satisfied with the application of the DD. In earlier studies, patients indicated that a DD was beneficial to both dentists and the patients [24,27]. The possible explanation for our results could be the information given to patients regarding DD use prior to the procedure. This is especially true with the fact that instrument inhalation is a life-threating accident [34,35] and is not uncommon [6,7,8,9,10,11,12,13]. This might also explain the positive attitude of patients because of the feeling of security due to the placement of a DD. Susini et al. reported that 36% of instrument ingestion cases required hospitalization [7]. This implies that nothing surpasses the safety of the DD while treating dental patients. The lowest proportion of patients highlighted the importance of DDs as a barrier in spreading infection. Patients may assume that cross-infection control policies are already well established and followed by the dental team, especially in governmental dental clinics. Nevertheless, the DD not only minimizes the risk of contamination of the patient’s root canal system by indigenous oral bacteria, but also protects the dentist and dental assistant against infections which can be transmitted by the patient’s saliva [3]. 

A previous study investigating the frequency of DD use in Saudi dental practice revealed that the majority of participants (90.7% endodontists and 74.1% general dental practitioners suggested a mandatory use policy of DDs in dental practice [17]. An almost similar trend was found in our study, as the second highest proportion of patients believed that DDs should be mandatory (32.2%). Patients were usually neither aware of regulations and bylaws of the dental profession, nor all aspects of DD placement for RCTs. This may explain why the highest proportion in our study (40.9%) reported that the decision to use DDs should be made by the dentist. However, this perception, to some extent, was limited to those who would prefer the placing of a DD in the next visit. The highest proportion of those who would not prefer the placing of a DD in the next visit believed that placing a DD should be based on patient preferences (51.6%). This trend within this group of patients was obvious when 9.1% of them reported that a DD is not needed.

The discrepancies between the current study and previous studies are based on specific aspects of patient acceptance of placing a DD during RCTs. This validates the conduction of our study and confirms our assumption, in the first instance, that different dental profession environments may affect the preferences, perceptions and opinions of patients on using DDs. However, there are certain limitations; this study only included patients attending the governmental sector clinics. Therefore, conducting further research including private sector patients is recommended. In addition, we believe that including additional centers from Middle-Eastern and European countries may show the real impact of various environmental, economic and cultural factors on patient preferences. Nevertheless, this study added further proof that patients, overall, have no objection towards the placing of a DD during RCTs. Hence, further research is needed in an attempt to close the gap in using DDs between the undergraduate training stage and post-graduation practice. 

## 5. Conclusions

Within the limitation of the current study, it can be concluded that patients generally accepted having a DD placed and were willing to undergo RCT procedures under DD isolation for future visits. Patient safety remains the most attractive advantage for patients to the application of DD use. Patient factors such as race, age, gender and level of education did not have any remarkable impact on patient preference in placing a DD during root canal treatment. Additionally, the time required to place a DD, first experience in placing a DD, treating clinician and sector of dental services seem to be the key factors influencing patient preferences.

## Figures and Tables

**Table 1:** Participant preferences for using a dental-dam (DD) in the next visit (%) and its correlation with participant countries of origin.

Participant Preference in Using a DD in the Next Visit	Countries of Origin					
	Saudi Arabia	Arab Asian	Arab African	Asian	African and Western Countries	Total *
Would Prefer	126 (85.1)	28 (80)	18 (78.3)	23 (69.7)	19 (90.5)	214 (81.6) *
Would not prefer	22 (14.9)	7 (20)	5 (21.7)	10 (30.3)	2 (9.5)	46 (18.4)
Total	148 [56.6] (100)	35 [14] (100)	23 [9.1] (100)	33 [12.5] (100)	21 [7.9] (100)	260 (100)

* The values in brackets [ ] and parentheses ( ) are the proportion of each country of origin’s category and the overall proportions of participants preference on using a DD in the next visit, respectively.

**Table 2:** The correlation of participant gender, age (years) and level of education with their preference on using a DD in the next visit ((%) and [%]).

**Participant Gender**	**Participant Age (Year, %)**					
	**15–20**	**21–30**	**31–40**	**41–50**	**Over 50**	**Total**
Male (45.2)	29 (23.6)	44 (35.8)	25 (20.3)	12 (9.8)	13 (10.6)	123 (100) [79.7] *
Female (54.8)	42 (29.8)	38 (27)	42 (29.8)	14 (9.9)	5 (3.5)	141 (100) [83.2]
Total	71 (26.8) [71.8]	82 (31.1) [89]	67 (25.4) [74.6]	26 (9.8) [84.6]	18 (6.8) [100]	264 (100) [81.6]
**Participant Preferences in Using a DD in the Next Visit**	**Participant Education Level (%)**					
	**Primary**	**Secondary**	**University**		**Postgraduate**	**Total**
Would prefer	40 (19.4)	98 (47.6)	57 (27.7)		11 (5.3)	206 (100)
Would not prefer	15 (30.6)	26 (53.1)	6 (12.2)		2 (4.1)	49 (100)
Total	55 (21.6)	124 (48.6)	63 (24.7)		13 (5.1)	255 (100)

* The values in brackets [ ] represent the proportions of participants who would prefer using a DD in the next visit.

**Table 3:** The correlation of preferring to use a DD in the next visit and the place that provided the endodontic treatment (%). TUCOD = Taibah University College of Dentistry. KFGH = King Fahad General Hospital.

Centre Providing Treatments	Clinicians Providing					
	Endo Consultant	Endo Postgrad or Demonstrator	Intern	General Dental Practitioners	Undergrad Students	Total
TUCOD (65.2)	36 (22.2)	23 (14.2)	36 (22.2)	0	67 (41.4)	162 (100) [74.4] *
KFGH (34.8)	42 (48.8)	1 (1.2)	9 (10.5)	34 (39.5)	0 (0)	86 (100) [95.7]
Total	78 (31.5) [93.2]	24 (9.7) [66.7]	45 (18.1) [86.7]	34 (13.7) [88.2]	67 (27) [61.2]	248 (100) [81.6]

* The values in brackets [ ] represent the proportions of participants who would prefer using a DD in the next visit.

**Table 4:** The correlation of participant preference in using a DD in the next visit and the DD advantages they reported (%).

Participant Preferences in Using a DD in the Next Visit	Easy Work	Irrigant Safe Use	Prevent File Swallowing	Better Cross Infection Control	Better Treatment Outcome	Other	Total
Would prefer	25 (11.4)	26 (11.8)	103 (46.8)	22 (10)	5 (2.3)	27 (17.7)	220 (100)
Would not prefer	12 (26.1)	2 (4.3)	18 (39.1)	0 (0)	7 (15.2)	5 (15.3)	46 (100)
Total	37 (13.7)	28 (11.9)	121 (44.8)	22 (8.1)	12 (4.4)	32 (17.1)	266 (100)

**Table 5:** The correlation between participant preferences in using a DD in the next visit and factors related to the current treatment session (time to place DD, duration of the current session, personal experience of the current session and dentist explanation to participants on reasons for DD usage).

**Participant Preferences in Using a DD in the Next Visit**	**Time (minutes) Required for Placing DD (%)**				
	**Up to 1**	**Up to 2**	**Up to 3**	**Over 3**	**Total**
Would prefer	169 (77.9)	22 (10.1)	2 (0.9)	24 (11.1)	217 (100)
Would not prefer	24 (52.2)	0	6 (13)	16 (34.8)	46 (100)
Total	193 (72.3)	22 (8.2)	8 (3)	40 (16.5)	263 (100)
**Participant Preferences in Using a DD in the Next Visit**	**Time Duration of Session with DD in Place (%)**				
	**Long**	**Short**		**Reasonable**	**Total**
Would prefer	41 (18.5)	23 (10.4)		158 (71.2)	222 (100)
Would not prefer	26 (52)	0 (0)		24 (48)	31 (100)
Total	67 (24.3)	23 (8.3)		182 (67.4)	272 (100)
**Participant Preferences in Using a DD in the Next Visit**	**Current Experience in Placing DD**				
	**Pleasant**	**Uncomfortable**	**Painful**	**No Comment**	**Total**
Would prefer	169 (76.8)	30 (13.6)	10 (4.5)	11 (5)	220 (100)
Would not prefer visit	7 (14)	35 (70)	5 (10)	3 (6)	50 (100)
Total	180 (65.7)	65 (23.7)	15 (5.5)	14 (5.1)	270 (100)
**Participant Preferences in Using a DD in the Next Visit**	**Did the Clinician Explain the Reasons for DD Use?**				
	**No**	**Yes in Details**		**Yes Briefly**	**Total**
Would prefer	60 (27.6)	87 (40.1)		70 (32.3)	217 (100)
Would not prefer	13 (26)	17 (34)		20 (40)	50 (100)
Total	73 (26.9)	104 (39.9)		90 (33.2)	267 (100)

**Table 6:** The correlation of participant opinion on the best policy of DD use and their preference in using a DD in the next visit (%).

Participant Preferences in Using a DD in the Next Visit	Policy for Using DD (%)				
	Mandatory	Optional (Up to Patient)	Optional (Up to Dentists)	Not Needed	Total
Would prefer	85 (38.3)	39 (17.6)	98 (44.1)	0 (0)	222 (100)
Would not prefer	5 (10)	26 (52)	14 (28)	5 (10)	50 (100)

## Appendix A

Survey Questionnaire

Treating Clinician:

- Tooth type (No)

Patient Information:

- Native spoken language: ▯ Arabic ▯ English ▯ Other:..............
- Any relevant or serious medical condition:...................................
- How old are you: ▯ 15–20 ▯ 20–30 ▯ 30–40 ▯ 40–50 ▯ Over 50
- Gender: ▯ Male ▯ Female
- Education level:……………………………………………

1. Have you ever had RCT before? ▯ Yes ▯ No

2. Have you ever experienced rubber dam placement?

   ▯ No ▯ Yes but not for RCT ▯ Yes for RCT

3. Compared to the previous experience, how was the current experience of DD placement?

   ▯ Almost the same ▯ Better ▯ Worse ▯ Cannot comment

4. Did the doctor explain the reasons for using a DD?

   ▯ Yes in detail (explanation was clear) ▯ Yes briefly (fair or poor) ▯ No

5. Time taken to apply the rubber dam:.................. minutes

6. Was the placing of the DD: ▯ pleasant (comfortable) ▯ uncomfortable ▯ painful ▯ cannot comment

7. How did you find the session with the DD in place?

   ▯ Long ▯ Short ▯ Reasonable ▯ Cannot comment

8. What is the most important advantage of using a DD?

   ▯ Aseptic working field ▯ Protection of soft tissue ▯ Better treatment quality

   ▯ Easy work for dentists and assistant (four-handed dentistry)

   ▯ Safe use of irrigants and medication ▯ Prevent swallowing of instruments and materials

   ▯ Better cross-infection control ▯ Better anticipated success rate of the treatment

9. What is the most important disadvantage of using a DD?

   ▯ Dribbling ▯ Hyper salivation ▯ Difficulty in swallowing or breathing

   ▯ Difficult communication with dentist during treatment ▯ Duration or process of placement

   ▯ Pain or discomfort while the DD is in place ▯ Impossibility of rinsing mouth during treatment

   ▯ Unpleasant taste during treatment ▯ Other: please specify:……………

10. Do you think that using a rubber dam should be?

  ▯ Not needed ▯ Optional, up to patient ▯ Optional, up to dentists ▯ Mandatory

11. Would you prefer using a DD in the next session? ▯ Yes ▯ No
